# Association between baseline serum glucose, triglycerides and total cholesterol, and prostate cancer risk categories

**DOI:** 10.1002/cam4.665

**Published:** 2016-02-29

**Authors:** Rhonda Arthur, Henrik Møller, Hans Garmo, Lars Holmberg, Pår Stattin, Håkan Malmstrom, Mats Lambe, Niklas Hammar, Göran Walldius, David Robinson, Ingmar Jungner, Mieke Van Hemelrijck

**Affiliations:** ^1^ Division of Cancer StudiesCancer Epidemiology GroupKing's College LondonLondonUnited Kingdom; ^2^ Regional Cancer CentreUppsalaSweden; ^3^ Department of Surgical SciencesUppsala University HospitalUppsalaSweden; ^4^ Departments of Surgical and Perioperative SciencesUrology and AndrologyUmeå UniversityFaculty of MedicineUppsalaSweden; ^5^ Unit of EpidemiologyInstitute of Environmental MedicineKarolinska InstitutetStockholmSweden; ^6^ Departments of Medical Epidemiology and BiostatisticsKarolinska InstitutetStockholmSweden; ^7^ AstraZeneca SverigeSödertaljeSweden; ^8^ Department of Cardiovascular EpidemiologyInstitute of Environmental MedicineKarolinska InstitutetStockholmSweden; ^9^ Department of Clinical Epidemiological UnitKarolinska Institutet and CALAB ResearchStockholmSweden

**Keywords:** Glucose, prostate cancer, total cholesterol, triglycerides

## Abstract

Lifestyle‐related risk factors such as hyperglycemia and dyslipidemia have been associated with several cancers. However, studies exploring their link with prostate cancer (PCa) clinicopathological characteristics are sparse and inconclusive. Here, we investigated the associations between serum metabolic markers and PCa clinicopathological characteristics. The study comprised 14,294 men from the Swedish Apolipoprotein MOrtality RISk (AMORIS) cohort who were diagnosed with PCa between 1996 and 2011. Univariate and multivariable logistic regression were used to investigate the relation between glucose, triglycerides and total cholesterol and PCa risk categories, PSA, Gleason score, and T‐stage. Mean age at time of PCa diagnosis was 69 years. Men with glucose levels >6.9 mmol/L tend to have PSA<4 *μ*g/L, while those with glucose levels of 5.6–6.9 mmol/L had a greater odds of PSA>20 *μ*g/L compared to PSA 4.0–9.9 *μ*g/L. Hypertriglyceridemia was also positively associated with PSA>20 *μ*g/L. Hyperglycemic men had a greater odds of intermediate‐ and high‐grade PCa and advanced stage or metastatic PCa. Similarly, hypertriglyceridemia was positively associated with high‐grade PCa. There was also a trend toward an increased odds of intermediate risk localized PCa and advanced stage PCa among men with hypertriglyceridemia. Total cholesterol did not have any statistically significant association with any of the outcomes studied. Our findings suggest that high serum levels of glucose and triglycerides may influence PCa aggressiveness and severity. Further investigation on the role of markers of glucose and lipid metabolism in influencing PCa aggressiveness and severity is needed as this may help define important targets for intervention.

## Introduction

An increasing number of studies suggest a role for lipid and glucose metabolism in prostate cancer (PCa) development. Findings from a recent meta‐analysis reported a strong positive association between obesity and risk of advanced prostate cancer 1, indicating that lifestyle‐related risk factors influence PCa aggressiveness and progression. Nonetheless, epidemiological evidence on the association between other lifestyle‐related risk factors, including dyslipidemia and diabetes, and PCa development and progression remains sparse and inconclusive 2, 3, 4, 5. Moreover, most of these studies are limited by few cases, short follow‐up time, and lack of power to detect the true associations between the exposures and the outcomes. Biologically, both dyslipidemia and hyperglycemia have been implicated with prostate carcinogenesis. Evidence from experimental studies using in vivo and in vitro models demonstrated that they may induce prostate carcinogenesis by modulating signaling pathways, which promote carcinogenic processes such as cell growth and proliferation, inflammation, oxidative stress, and cell migration 6, 7, 8, 9, 10, 11, 12, 13, 14, 15.

Based on data in the AMORIS cohort, a large Swedish database with information on over 800,000 men and women, we have previously identified that abnormal serum lipid and glucose profiles may be involved in risk of developing PCa. Our findings showed a positive association between hypertriglyceridemia and PCa risk in hyperglycemic men 16. Conversely, we found that high‐density lipoprotein and Apolipoprotein A‐1 were inversely associated with PCa risk 17. Here, we further investigated this by evaluating the association between serum total cholesterol, triglycerides and glucose and PCa risk categories and clinicopathological characteristics (i.e., PSA, Gleason score, and TNM staging) in the updated AMORIS cohort.

## Materials and Methods

### Study design and population

Detailed description of the AMORIS cohort can be found elsewhere 16, 18. Briefly, this database comprises 812,073 Swedish men and women with blood samples sent for laboratory evaluation to the Central Automation Laboratory (CALAB) in Stockholm, Sweden, during the period 1985–1996 19, 20, 21. Individuals recruited were primarily from the greater Stockholm area, who were either healthy and having laboratory testing as part of a general checkup or outpatients referred for laboratory testing. None of the participants were inpatients at the time of sampling. In the AMORIS cohort, the CALAB database was linked to several Swedish national registries such as the Swedish National Cancer Register, the Hospital Discharge Register, the Cause of Death Register, the consecutive Swedish Censuses during 1970–1990, and the National Register of Emigration using the Swedish 10‐digit personal identity number.

For this study, we specifically focused on the linkage between the AMORIS database and the National Prostate Cancer Register (NPCR), which has been nationwide since 1998 22. NPCR was developed to provide data for quality assurance and includes 98% of all newly diagnosed PCa cases registered in the Swedish National Cancer Register 22 to which reporting is mandated. From the NPCR, we extracted information on date of diagnosis, age at diagnosis, TNM stage 19, Gleason score, serum concentration of PSA at time of diagnosis, and primary treatment given or planned up to 6 months after date of diagnosis. Information on educational level was retrieved from the Population and Housing Census for 1970–1990. Using information from the National Patient Register, we calculated the Charlson comorbidity index which includes 19 diseases, with each disease category assigned a weight. The sum of an individual's weights was used to create a score, resulting in four comorbidity levels ranging from no comorbidity to severe comorbidity (0, 1, 2, and ≥3) 23.

From AMORIS, we selected all men aged 20 and older diagnosed with PCa (and information on their prostate tumor characteristics from 1996 until 31 December 2011), who had baseline levels of serum glucose, total cholesterol, and triglycerides taken from the same health visit 19. A total of 14,294 men were included in the final analysis. Figure S1 provides an overview of the cohort selection process.

Total cholesterol and triglycerides were measured enzymatically as previously described 24. Glucose was also measured enzymatically with a glucose oxidase/peroxidase method. All methods were fully automated with automatic calibration and performed at one accredited laboratory 20.

The primary endpoints for our study are PCa risk categories. These risk categories were defined in accordance with an adapted version of National Comprehensive Cancer Network as low risk (T1‐2, Gleason score 2–6 and PSA <10 ng/mL), intermediate risk (T1‐2, Gleason score 7 and/or PSA 10 to <20 ng/mL), high risk (T3 and/or Gleason score 8–10 and/or PSA 20 to <50 ng/mL), and regionally metastatic tumors (T4 and/or N1 and/or PSA 50–<100 ng/mL in the absence of distant metastases (M0 or MX)) and distant metastatic tumors (M1 and/or PSA >100 ng/mL) 22. We also included the clinicopathological characteristics (PSA levels, Gleason score, T stage) as outcomes. PSA was categorized as <4 *μ*g/ L, 4–9.9 *μ*g/ L, 10–20 *μ*g/L and >20 *μ*g/L. Gleason score was defined as low‐grade tumor (Gleason score ≤6 or WHO grade 1), intermediate grade tumor (Gleason score 7 or WHO grade 2) and high‐grade tumor (Gleason score ≥8 or WHO grade 3), while T stage was defined as localized (T1–T2) and advanced (T3–T4).

### Statistical analysis

Odds ratios and 95% confidence intervals for the association between serum glucose, total cholesterol and triglycerides and PCa clinicopathological characteristics were calculated using logistic regression. In multivariable analyses, models were adjusted for age at diagnosis, educational level (low, intermediate, high), CCI (0, 1, 2, ≥3), and fasting status (fasting, nonfasting, missing). For each man, we also calculated the interval time between the time from blood analyses and date of PCa diagnosis, which was also included as a covariate in the model. All models were also mutually adjusted for serum glucose, total cholesterol, and triglycerides. Due to the high percentage of cases with missing information on body mass index (BMI) (78.5%), BMI was not included as a covariate in our multivariate model. These missing value indicators were included in the statistical models. We did conduct a sensitivity analysis in the subgroup with information on BMI (*n* = 3073), whereby we adjusted for BMI. Due to the small sample size, statistical significance disappeared, but the directions of the associations virtually remained the same. We also conducted stepwise regression analyses among those with information on BMI to assess the effect of excluding BMI on the multivariate model. The results of our stepwise regression analyses showed that adjusting for BMI had little or no effect on the multivariate model, thus suggesting that BMI may not be an important confounder in this study population. Hence, all results shown are focused on the cohort of 14,294 men. Missing values were assigned to separate categories for education (1.5%) and fasting status (11.0%).

Serum glucose, total cholesterol, and triglycerides levels were analyzed using clinical cut‐offs in accordance with the American Diabetes Association and National Cholesterol Education Programme (NCEP) guidelines^25^, ^26^, ^27^. Serum glucose levels were categorized as <5.6 mmol/L, 5.6–6.9 mmol/L, and >6.9 mmol/L, while serum total cholesterol was classified as <5.18 mmol/L, 5.18 mmol/L–6.19 mmol/L, and >6.19 mmol/L and serum triglycerides as <1.7 mmol/L, 1.7–2.24 mmol/L, and >2.24 mmol/L. The lowest clinical cut‐offs were selected as the reference category.

Finally, we conducted a sensitivity analysis whereby we excluded men with measurements taken 2 years or less prior to the date of PCa diagnosis to assess possible reverse causation. We also performed a sensitivity analysis whereby we excluded men with nonfasting glucose measurements.

Data management and statistical analyses were conducted with Statistical Analysis Software (SAS) release 9.4 (SAS Institute, Cary, NC).

## Results

Table 1 illustrates the baseline characteristics of the study population. Mean age at PCa diagnosis was 69 ± 8.0 years. The median levels of glucose, total cholesterol, and triglycerides at baseline were 4.9 mmol/L, 5.8 mmol/L, and 1.2 mmol/L, respectively. Most men had normal glucose (<5.60 mmol/L) and triglycerides (<1.7 mmol/L) levels (82.5% and 69.1%, respectively), while at least 75% men had borderline high or high total cholesterol levels with mean time between measurement and PCa diagnosis of 16.7 ± 4.8 years.

**Table 1:** Baseline characteristics of study population

Characteristics	Total population (*N* = 14294)
Age at diagnosis (years)	
Mean (SD)	69 (8.0)
*N* (%)	
≤49	95 (0.7)
50–59	1971 (13.8)
60–69	6321 (44.2)
≥70	5916 (41.4)
Education	
High	4427 (31.0)
Intermediate	5912 (41.2)
Low	3740 (26.2)
Missing	215 (1.5)
Charlson comorbidity index	
0	10651 (74.5)
1	1840 (12.9)
2	1104 (7.7)
≥3	699 (4.9)
Glucose (mmol/L)	
Median (IQR)	4.9 (4.6–5.4)
*N* (%)	
<5.60	11796 (82.5)
5.6–6.9	2062 (14.4)
>6.9	436 (3.1)
Total cholesterol (mmol/L)	
Median (IQR)	5.8 (5.2–6.5)
*N*(%)	
<5.18	3552 (24.9)
5.18–6.19	5394 (37.7)
>6.19	5348 (37.4)
Triglycerides (mmol/L)	
Median (IQR)	1.2 (0.9–1.8)
*N* (%)	
<1.70	9878 (69.1)
1.70–2.24	2199 (15.4)
≥2.25	2217 (15.5)
Fasting status	
*N* (%)	
Fasting	8577 (60.0)
Nonfasting	4143 (29.0)
Missing/Unknown	1574 (11.0)
Interval time between measurement and prostate cancer diagnosis	
Mean (SD)	16.7 (4.8)
PSA (*μ*g/L)	
Median (IQR)	9.4 (5.8–20.0)
*N*(%)	
>4	1164 (8.1)
4.0–9.9	6126 (42.9)
10.0–20.0	3219 (22.5)
>20.0	3351 (23.4)
Missing	434 (3.0)
Gleason grade	
*N* (%)	
Low	6942 (48.6)
Intermediate	4789 (33.5)
High	2320 (16.2)
Missing	243 (1.7)
Clinical stage	
*N* (%)	
T1	6797 (47.6)
T2	4290 (30.0)
T3	2496 (17.5)
T4	319 (3.2)
Missing	392 (2.7)
N0	1502 (10.5)
N1	292 (1.0)
Missing/NX	12500 (87.5)
M0	4665 (32.6)
M1	867 (6.1)
Missing/MX	8762 (61.3)
Risk categories	
*N* (%)	
Localized	
Low risk	4472 (31.3)
Intermediate risk	4032 (28.2)
High risk	3258 (22.8)
Regional/distant metastatic	2187 (15.3)
Missing	345 (2.4)

Men with glucose in the prediabetic range (5.6–6.9 mmol/L) had a greater odds of high risk and metastatic cancer, as compared to normoglycemic men (OR: 1.13; 95% CI: 0.98–1.30 and OR: 1.21; 95% CI: 1.04–1.42 for high risk and metastatic PCa, respectively) (Table 2). Triglycerides were positively associated with intermediate risk localized PCa for the highest versus the lowest subgroup (OR: 1.17; 95% CI: 1.03–1.34). Positive but nonstatistically significant associations between hypertriglyceridemia and high risk and metastatic PCa were also observed. When considering total cholesterol, there was no evidence to suggest any differences in any of the outcomes studies based on serum total cholesterol levels.

**Table 2:** Odds ratios (OR) and 95% CI for risk categories (intermediate‐risk localized/low‐risk localized, high‐risk localized/low‐risk localized and metastatic/low‐risk localized) by baseline levels of serum glucose, total cholesterol, and triglycerides

	Glucose (mmol/L)^a^			*P* for trend	Total cholesterol (mmol/L)			*P* for trend	Triglycerides (mmol/ L)			*P* for trend
	<5.60	5.60–6.90	>6.90		<5.18	5.18–6.19	>6.19		<1.70	1.70–2.24	≥2.25	
Low risk (Reference level for outcome studied)												
*N* = 4472	3794	558	120		1189	1689	1594		3152	690	630	
Intermediate risk												
*N* = 4032	3359	566	107		1029	1527	1476		2784	603	645	
Age‐adjusted OR (95% CI)	1.00 (Ref)	1.08 (0.95–1.23)	0.94 (0.72–1.22)	0.601	1.00 (Ref)	0.97 (0.87–1.08)	0.96 (0.86–1.07)	0.469	1.00 (Ref)	0.99 (0.87–1.11)	1.17 (1.03–1.32)	0.030
Multivariable adjusted OR (95% CI)	1.00 (Ref)	1.07 (0.94–1.21)	0.90 (0.69–1.19)	0.864	1.00 (Ref)	0.96 (0.86–1.07)	0.93 (0.83–1.05)	0.228	1.00 (Ref)	0.99 (0.88–1.12)	1.17 (1.03–1.34)	0.037
High risk												
*N* = 3258	2630	523	105		752	1216	1290		2216	529	513	
Age–adjusted OR (95% CI)	1.00 (Ref)	1.23 (1.07–1.41)	1.09 (0.82–1.44)	0.016	1.00 (Ref)	1.00 (0.88–1.13)	1.06 (0.94–1.20)	0.287	1.00 (Ref)	1.09 (0.96–1.25)	1.20 (1.05–1.37)	0.006
Multivariable adjusted OR (95% CI)	1.00 (Ref)	1.13 (0.98–1.30)	0.93 (0.69–1.24)	0.394	1.00 (Ref)	0.97 (0.85–1.10)	0.99 (0.87–1.13)	0.935	1.00 (Ref)	1.05 (0.92–1.21)	1.14 (0.98–1.31)	0.083
Regional/distant metastatic												
*N* = 2187	1726	371	90		506	822	859		1487	323	377	
Age‐adjusted OR (95% CI)	1.00 (Ref)	1.32 (1.14–1.54)	1.43 (1.06–1.92)	<0.001	1.00 (Ref)	1.01 (0.88–1.17)	1.05 (0.91–1.21)	0.449	1.00 (Ref)	0.97 (0.83–1.13)	1.31 (1.12–1.52)	0.003
Multivariable adjusted OR (95% CI)	1.00 (Ref)	1.21 (1.04–1.42)	1.10 (0.80–1.49)	0.048	1.00 (Ref)	0.97 (0.83–1.12)	0.95 (0.82–1.10)	0.515	1.00 (Ref)	0.92 (0.78–1.07)	1.17 (0.99–1.37)	0.172

Multivariable models‐adjusted for age educational level, Charlson comorbidity index, serum glucose, total cholesterol, triglycerides, fasting status, time between measurement, and prostate cancer diagnosis.

Ref, Reference.

a Not adjusted for Charlson comorbidity index.

When studying PSA as an outcome, we observed that a greater proportion of men with glucose levels >6.90 mmol/L had PSA <4 *μ*g/L (OR: 1.43; 95% CI: 1.01–1.45), compared to PSA levels of 4.0–9.9 *μ*g/L. There was also a positive association between glucose levels of 5.6–6.9 mmol/L and PSA levels >20 *μ*g/L compared to PSA levels of 4.0–9.9 *μ*g/L (OR: 1.28; 95% CI: 1.13–1.45) (Table 3). Moreover, we found a positive association between triglycerides ≥2.25 mmol/L and PSA levels >20 *μ*g/L (OR: 1.17; 95% CI: 1.03–1.34), as compared to PSA levels of 4.0–9.9 *μ*g/L.

**Table 3:** Odds ratio and 95% CI for the association between serum lipids (total cholesterol and triglycerides) and glucose levels and PSA levels and Gleason score

	Glucose (mmol/L)^a^			*P* for trend	Total cholesterol (mmol/L)			*P* for trend	Triglycerides (mmol/L)			*P* for trend
	<5.60	5.60–6.90	>6.90		<5.18	5.18–6.19	>6.19		<1.70	1.70–2.24	≥2.25	
PSA <4 versus 4.0–9.9(*μ*g/L)	971	152	41		329	448	387		816	188	160	
*N* = 6126												
Age‐adjusted OR (95% CI)	1.00 (Ref)	1.06 (0.88–1.28)	1.41 (1.00–2.01)	0.081	1.00 (Ref)	0.99 (0.86–1.01)	0.84 (0.99–1.16)	0.105	1.00 (Ref)	1.06 (0.89–1.26)	0.94 (0.78–1.13)	0.698
Multivariable adjusted OR (95% CI)	1.00 (Ref)	1.08 (0.89–1.30)	1.43 (1.01–2.05)	0.071	1.00 (Ref)	0.99 (0.85–1.16	0.88 (0.75–1.05)	0.130	1.00 (Ref)	1.09 (0.91–1.30)	0.98 (0.80–1.18)	0.900
PSA 4.0–9.9 (*μ*g/L) (Reference level for outcome studied)												
*N* = 1164	5184	781	161		1591	2279	2256		4292	937	897	
PSA 10.0–20.0 versus 4.0–9.9(*μ*g/L)												
*N* = 3219	2684	442	93		749	1253	1217		2205	507	507	
Age‐adjusted OR (95% CI)	1.00 (Ref)	1.05 (0.92–1.19)	1.04 (0.80–1.35)	0.485	1.00 (Ref)	1.09 (0.98–1.22)	1.03 (0.92–1.16)	0.754	1.00 (Ref)	1.05 (0.93–1.19)	1.13 (1.00–1.28)	0.044
Multivariable adjusted OR (95% CI)	1.00 (Ref)	0.98 (0.85–1.11)	0.91 (0. 69–1.18)	0.450	1.00 (Ref)	1.05 (0.94–1.18)	0.98 (0.87–1.10)	0.576	1.00 (Ref)	1.04 (0.92–1.18)	1.11 (0.97–1.26)	0.119
PSA >20.0 versus 4.0–9.9(*μ*g/ L)												
*N* = 3351	2625	595	131		789	1232	1330		2284	505	562	
Age‐adjusted OR (95% CI)	1.00 (Ref)	1.41 (1.25–1.60)	1.41 (1.10–1.81)	<0.001	1.00 (Ref)	0.97 (0.86–1.09)	1.00 (0.89–1.13)	0.889	1.00 (Ref)	1.01 (0.89–1.14)	1.22 (1.08–1.38)	0.004
Multivariable adjusted OR (95% CI)	1.00 (Ref)	1.28 (1.13–1.45)	1.06 (0.82–1.37)	0.004	1.00 (Ref)	0.92 (0.82–1.04)	0.91 (0.81–1.03)	0.153	1.00 (Ref)	0.98 (0.86–1.11)	1.17 (1.03–1.34)	0.040
Gleason ≤6 (Reference level for outcome studied)		
*N* = 6942	5859	896	187		1175	2637	2530		4841	1059	1042	
Gleason 7 versus ≤6												
*N* = 4789	3913	727	149		1195	1795	1799		3314	733	742	
Age‐adjusted OR (95% CI)	1.00 (Ref)	1.14 (1.01–1.27)	1.04 (0.81–1.33)	0.076	1.00 (Ref)	0.95 (0.86–1.06)	0.97 (0.87–1.07)	0.588	1.00 (Ref)	1.02 (0.91–1.14)	1.06 (0.95–1.18)	0.319
Multivariable adjusted OR (95% CI)	1.00 (Ref)	1.18 (1.05–1.33)	1.09 (0.84–1.40)	0.017	1.00 (Ref)	0.97 (0.87–1.07)	0.98 (0.88–1.08)	0.690	1.00 (Ref)	1.03 (0.92–1.17)	1.07 (0.95–1.20)	0.264
Gleason ≥8 versus ≤6												
*N* = 2320	1830	398	92		514	869	937		1557	367	396	
Age‐adjusted OR (95% CI)	1.00 (Ref)	1.26 (1.09–1.46)	1.39 (1.03–1.87)	<0.001	1.00 (Ref)	1.06 (0.92–1.22)	1.14 (1.00–1.31)	0.052	1.00 (Ref)	1.10 (0.95–1.28)	1.25 (1.09–1.45)	0.002
Multivariable adjusted OR (95% CI)	1.00 (Ref)	1.24 (1.07–1.44)	1.37 (1.02–1.87)	0.001	1.00 (Ref)	1.05 (0.91–1.21)	1.11 (0.96–1.28)	0.156	1.00 (Ref)	1.06 (0.92–1.24)	1.17 (1.01–1.37)	0.040
T1–T2 (Reference level for outcome studied)												
*N* = 11087	9256	1525	306		2814	4197	4076		7725	1681	1681	
T3–T4 versus T1–T2												
*N* = 2815	2226	474	115		656	1040	1119		1888	455	472	
Age‐adjusted OR (95% CI)	1.00 (Ref)	1.21 (1.08–1.36)	1.42 (1.13–1.77)	<0.001	1.00 (Ref)	0.98 (0.88–1.10)	1.04 (0.94–1.17)	0.367	1.00 (Ref)	1.12 (0.99–1.26)	1.18 (1.05–1.33)	0.002
Multivariable adjusted OR (95% CI)	1.00 (Ref)	1.12 (0.99–1.26)	1.20 (0.96–1.52)	0.022	1.00 (Ref)	0.95 (0.85–1.06)	0.98 (0.88–1.10)	0.860	1.00 (Ref)	1.08 (0.96–1.22)	1.12 (0.99–1.27)	0.046

Multivariable models–adjusted for age, educational level, Charlson comorbidity index, serum glucose, total cholesterol, triglycerides, fasting status, time between measurement, and prostate cancer diagnosis.

Ref, Reference.

a Not adjusted for Charlson comorbidity index.

For Gleason score, we found a positive association between glucose levels of 5.6–6.9 mmol/L and risk of intermediate‐grade (OR: 1.18; 95% CI: 1.05–1.33) and high‐grade PCa (OR: 1.24; 95% CI: 1.07–1.44) (Table 3). Compared to men with glucose <5.6 mmol/L, there was also an increased odds of high‐risk PCa among men with glucose levels in the diabetic range (>6.9 mmol/L) (OR: 1.37; 95%: 1.02–1.87). Hypertriglyceridemia was positively associated with high‐grade PCa (OR: 1.17; 95% CI: 1.01–1.37), but the odds of intermediate grade PCa did not appear to be different by triglycerides levels. Although neither glucose nor triglycerides had a statistically significant association with stage of PCa, there was an observed trend toward increased odds of T3–T4 PCa with increasing glucose and triglycerides levels (P for trend: 0.022 and 0.046, respectively).

The exclusion of 21 men with measurements taken less than 2 years prior to PCa diagnosis did not result in changes in the findings (Table S2 and S3). When we restricted our analyses to men with fasting glucose measurements, the direction of the associations remained virtually unchanged (Table S4).

## Discussion

In this study, we found evidence suggesting an independent positive association between serum glucose levels and higher grade (Gleason score 7 or ≥8) and advanced PCa (T3–T4, metastatic PCa). Similarly, men with hypertriglyceridemia (≥2.25 mmol/L) had increased odds of higher grade or more advanced PCa. When PSA was taken into account, we observed that men with glucose levels in the diabetic range (>6.9 mmol/L) had greater odds of having PSA levels <4 *μ*g/L, while those with glucose levels in the prediabetic range (5.6–6.9 mmol/L) had greater odds of having PSA >20 *μ*g/L. Men with hypertriglyceridemia (≥2.25 mmol/L) also had increased odds of PSA >20 *μ*g/L. There was, however, no evidence to suggest that the odds of any of the outcomes studied varied by total cholesterol levels.

### Glucose

**Figure 1:**
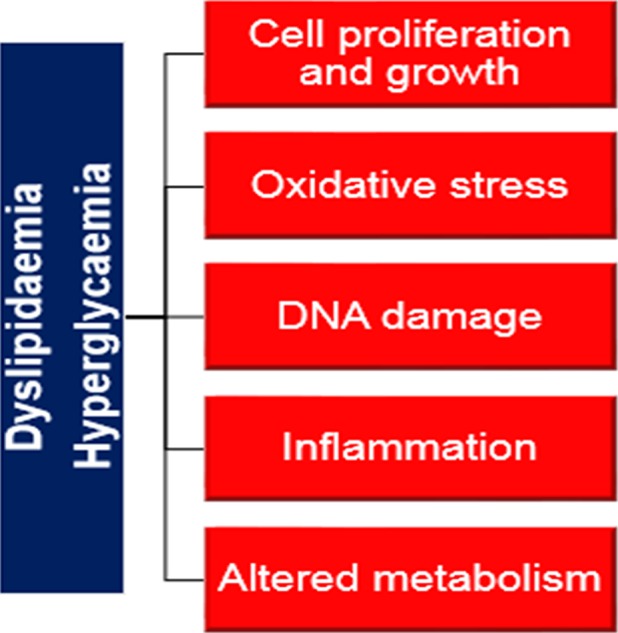
Schematic representation of carcinogenic processes suggested to be associated with hyperglycemia and dyslipidemia.

Hyperglycemia has been positively associated with cancers such as pancreatic, breast, and colorectal cancer 28, 29. However, its link with prostate carcinogenesis is conflicting. Consistent with our findings, four other studies found evidence of higher risk of more aggressive or advanced PCa among men with abnormal glucose levels with the association being nonsignificant in two of the studies 3, 4, 30, 31, 32. Conversely, several other studies reported a protective effect of hyperglycemia or diabetes against higher grade or more advanced PCa 33, 34, 35, 36, 37, 38. The observed positive association reported by our study may be explained by several underlying biological mechanisms. For several decades, glucose has been documented as an important source of energy for rapid tumor cell proliferation 39, 40. Evidence from clinical and genetic studies have also linked the hyperglycemic environment to carcinogenic processes such as apoptosis, oxidative stress, DNA damage, and chronic inflammation, which may drive the aggressiveness and progression of cancer (Fig. 1) 11, 12, 13, 14, 40, 41. For instance, one mice study found that translation of the glycolytic enzyme hexokinase 2 (HK2) was increased in PCa cells due to loss of Pten and p53, which help to prevent cells from growing uncontrollably 11. GLUT12, an important protein in the glycolytic pathway, was also observed to be highly expressed by PCacells and may potentially help to facilitate the high energy needs of tumor cells 12. Other metabolic changes such as hyperinsulinemia, increased level of bioavailable IGF 1, and increased production of advanced glycation end products (AGE), which occur in response to a hyperglycemic environment, have also been linked to increased proliferation of cancer cells and poorer cancer outcomes 42, 43.

Furthermore, the observed positive association between glucose and more aggressive or advanced PCa persisted even after correcting for the potential confounding effects of BMI. Unlike most of the observational studies which examined the link between diabetes and PCa outcomes, we focused on serum glucose which has been suggested to be more sensitive than diabetes for accurately determining the relationship between glucose regulation and disease outcomes 44. Nevertheless, in our study, we lacked information on duration of glucose aberrations. Prolonged glucose impairment has been reported to cause destruction of the Leydig cells, thereby, resulting in lower testosterone levels and potentially lowering the risk of worse outcomes 40, 45, 46, 47, 48. Nonetheless, evidence linking lower testosterone to reduced risk of worse PCa outcomes remains conflicting 46, 47, 48.

Similar to our study, one recent study reported higher PSA levels among prediabetic men 49. In line with our findings, several studies also reported that diabetes or glucose in the diabetic range was associated with lower PSA levels 50, 51, 52, 53, 54. Overall, our findings suggest that the effect of glucose levels in the prediabetic phase on PSA levels may be different from the diabetic phase. The potential mechanism through which prediabetes or impaired glucose tolerance may affect PSA levels is unknown. It is possible that in the prediabetic spectrum, the hormonal milieu characterized by hyperinsulinemia or higher IGF‐1 activity may play a role 41. With regard to men with glucose in the diabetic range, the underlying mechanisms which may partly explain the observed lower PSA levels involve reduction in testosterone levels and insulin‐growth factor‐1 bioactivity, resulting from hypoinsulinemia in the long term, and the use of metformin, a potential antineoplastic treatment 40, 41, 55, 56, 57. Additionally, obesity, which is common among men with abnormal glucose levels, may also have contributed to the observed lower PSA levels due to PSA hemodilution 40, 52, 58. However, in our subgroup analyses involving men with information on BMI, adjusting for BMI had minimal or no effect on the multivariate model.

Interestingly, we also found that although among men with glucose in the diabetic range (>6.9 mmol/L) had lower PSA levels, they had a greater odds of high‐grade or more advanced PCa. This finding may be partly due to the fact that men with PSA <4 *μ*g/L are not likely to be biopsied and may therefore be diagnosed in the symptomatic stage when the cancer is more aggressive or advanced. These results are also in line with other studies indicating that men with low‐PSA producing cancers tend to develop very poorly differentiated or highly tumorigenic castration‐resistant PCa cells 59, 60, 61.

### Total cholesterol and triglycerides

Four prospective studies found positive associations between total cholesterol and higher grade or more advanced PCa 5, 62, 63, 64, 65. Comparable to our study, the effect was positive, but statistically significant in two of these studies 5, 65. Upon adjustment for age, we observed that the statistical significant ORs disappeared, which is consistent with the well‐established view that age is a shared risk factor for both hypercholesterolemia and cancer 45, 66, 67.

Previously, we provided evidence supporting a potential role for lipid metabolism in PCa development 16, 17. Here, we provided further evidence showing that triglycerides may influence the aggressiveness and severity of PCa. Except for one small Japanese study, most epidemiological studies did not report a link between triglycerides and PCa prognostic outcomes 2. However, experimental studies using in vitro models, have shown that triglyceride‐rich remnant like particles induce carcinogenesis by upregulating cell signaling pathways, such as the MEK/ERK and Akt pathways, involved in controlling cell growth and proliferation, apoptosis, cell cycle arrest, and lipid biosynthesis (Fig 1) 7, 8, 68.

Consistent with results from previous studies, total cholesterol did not appear to have an influence on PSA levels 69, 70. In contrast to our findings, one study including 6774 Chinese men, reported an inverse association between triglycerides and PSA levels 71. Two other studies, however, did not find any association between triglycerides and PSA 69, 70. Further studies are needed to assess the relation between these lipids and PSA levels in men with PCa, as most studies to date involve healthy men from a relatively low‐risk Asian population.

### Strengths and limitations

This is one of the largest prospective studies to investigate the relation between glucose and lipid metabolism and clinicopathological characteristics of PCa. Our study population was also selected from an almost complete population‐based PCa register. Another strength of our study is the use of prediagnostic exposure measurements, which were all measured at a central laboratory. This enabled us to assess relations between the exposures and outcomes temporally and minimized the potential influence of reverse causation. Moreover, the results of our sensitivity analyses did not indicate any evidence of reverse causation.

PCa is a slow‐progressing cancer 72 with a long induction period estimated at 6–13 years by some studies 73. In this study, the relatively long time of 16.7 years between exposure measurement and PCa diagnosis, which may reflect the induction period between the exposures and PCa, can also be considered as a strength. However, we only used a single baseline exposure measurement, precluding consideration of the effect of longitudinal changes in exposure level over time 33, 37. Further studies using repeated measurements are therefore warranted to account for the potential effect of this limitation. Our study also lacks information on participants’ diabetic status and history of use of metformin and/or statin, so that exposure misclassification may have occurred. However, any such misclassification would be nondifferential since we used prediagnostic baseline exposure measurements. We did not have information on life‐style‐related factors such as smoking status and dietary history. Nonetheless, existing evidence does not appear to substantiate a link between the afore‐mentioned lifestyle risk factors and poorer PCa outcomes 1. Based on the results of our sensitivity analyses, the lack of information on fasting status for some men did not appear to influence our results. Finally, we lack follow‐up information on the clinicopathological characteristics of PCa such as PSA, and therefore, could not evaluate the association between the studied serum biomarkers and PCa biochemical recurrence or PCa progression.

## Conclusion

Our findings suggest that the potential influence of glucose on PSA levels may vary as one progress from the prediabetic phase to the diabetic phase. Interestingly, though men in both the intermediate and highest glucose subgroups had poorer PCa outcomes. These findings may have important clinical implications for PCa detection and treatment, particularly among men with high glucose and low PSA levels, as previous evidence have shown that low PSA level is also concomitant with poorer PCa survival 61. Overall, our findings further support a potential role for the lipid and glucose metabolism in prostate carcinogenesis.

## Conflict of Interests

None declared.

## Supporting information


**Figure S1.** Schematic representation of study population.

**Table S1.** Odds ratios (OR) and 95% CI for prostate cancer risk categories by baseline levels of serum glucose, total cholesterol, and triglycerides for men with measurements more than 2 years prior to diagnosis.
**Table S2**. Odds ratio and 95% CI for the association between serum lipids (total cholesterol and triglycerides) and glucose levels and PSA levels and Gleason score.
