# Supplementary figures and images for: Association between baseline serum glucose, triglycerides and total cholesterol, and prostate cancer risk categories
Source: Cancer Med. 2016 Feb 29;5(6):1307–18. doi: 10.1002/cam4.665 (PMC4924389; doi:10.1002/cam4.665)

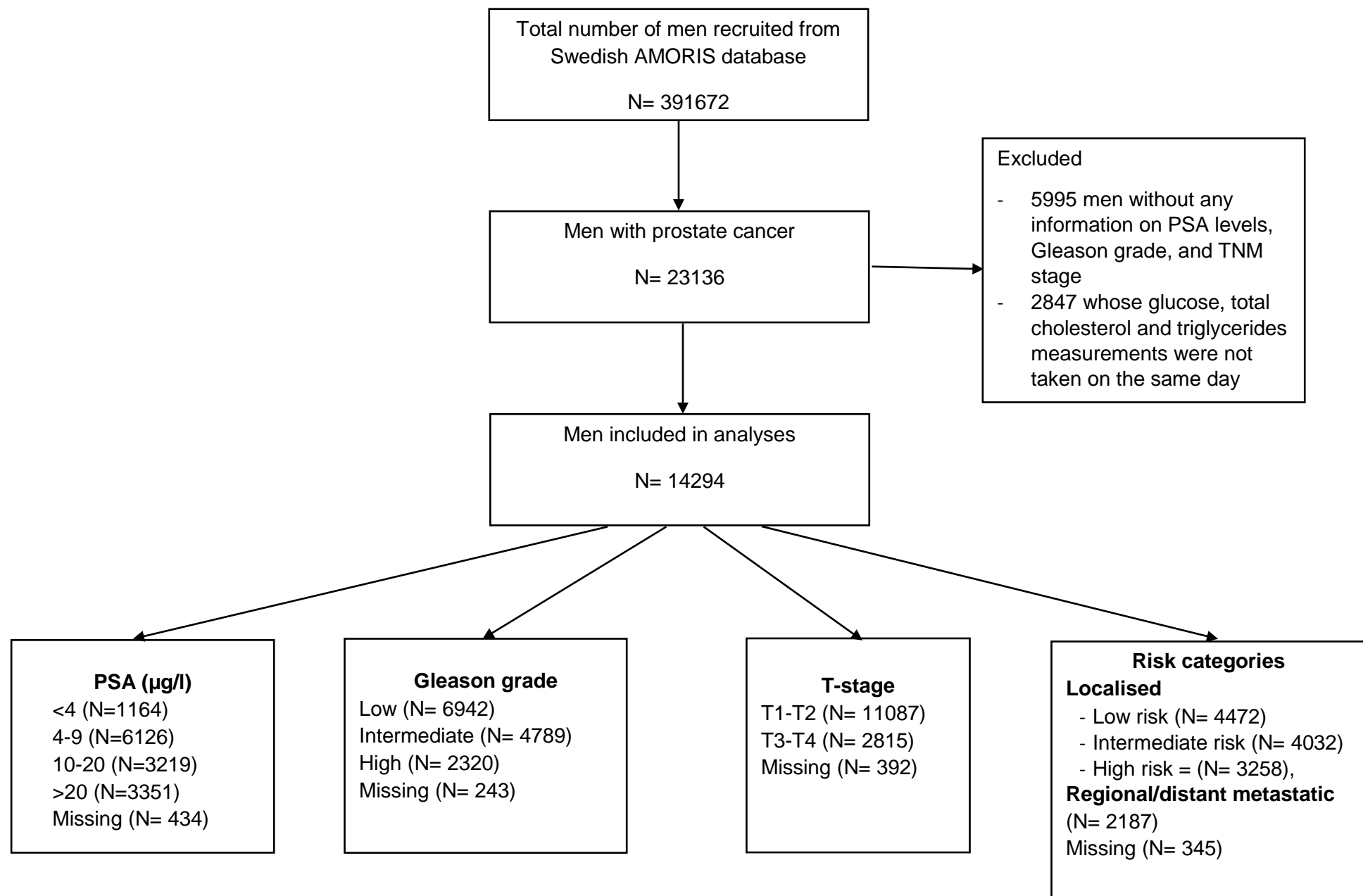

**Figure S1: Schematic representation of study population**

Supplement: Supplementary file 1 — Figure S1. Schematic representation of study population. [file CAM4-5-1307-s001.pdf]
