# Supplementary material for: Association between baseline serum glucose, triglycerides and total cholesterol, and prostate cancer risk categories
Source: Cancer Med. 2016 Feb 29;5(6):1307–18. doi: 10.1002/cam4.665 (PMC4924389; doi:10.1002/cam4.665)
Supplement: Supplementary file 2 — Table S1. Odds ratios (OR) and 95% CI for prostate cancer risk categories by baseline levels of serum glucose, total cholesterol, and triglycerides for men with measurements more than 2 years prior to diagnosis. Table S2. Odds ratio and 95% CI for the association between serum lipids (total cholesterol and triglycerides) and glucose levels and PSA levels and Gleason score. [file CAM4-5-1307-s002.docx]

**Supplementary results**

|  | **Glucose (mmol/l)*** | | | | **Total cholesterol (mmol/l)** | | |  | **Triglycerides (mmol/l)** | | |  |
| --- | --- | --- | --- | --- | --- | --- | --- | --- | --- | --- | --- | --- |
| **Prostate cancer risk categories** | **<5.60** | **5.60-6.90** | **>6.90** | **P for trend** | **<5.18** | **5.18-6.19** | **>6.19** | **P for trend** | **<1.70** | **1.70-2.24** | **≥2.25** | **P for trend** |
| **Low risk localised**  (Reference level for outcome studied) |  |  |  |  |  |  |  |  |  |  |  |  |
|  |  |  |  |  |  |  |  |  |  |  |  |  |
| **Intermediate risk localised**^¥^ |  |  |  |  |  |  |  |  |  |  |  |  |
| Age-adjusted OR (95% CI) | 1.00 (Ref) | 1.08 (0.95-1.22) | 0.93 (0.71-1.21) | 0.657 | 1.00 (Ref) | 0.98 (0.87-1.09) | 0.96 (0.86-1.08) | 0.493 | 1.00 (Ref) | 0.99 (0.87-1.11) | 1.17 (1.04-1.33) | 0.028 |
| Multivariate adjusted OR (95% CI) | 1.00 (Ref) | 1.06 (0.93-1.21) | 0.89 (0.68-1.17) | 0.924 | 1.00 (Ref) | 0.97 (0.87-1.09) | 0.93 (0.83-1.05) | 0.245 | 1.00 (Ref) | 0.99 (0.87-1.12) | 1.19 (1.04-1.35) | 0.023 |
|  |  |  |  |  |  |  |  |  |  |  |  |  |
| **High risk localised**^¥^ |  |  |  |  |  |  |  |  |  |  |  |  |
| Age-adjusted OR (95% CI) | 1.00 (Ref) | 1.23 (1.07-1.41) | 1.08 (0.82-1.44) | 0.017 | 1.00 (Ref) | 1.00 (0.88-1.13) | 1.07 (0.94-1.21) | 0.278 | 1.00 (Ref) | 1.09 (0.95-1.25) | 1.20 (1.04-1.37) | 0.007 |
| Multivariate-adjusted OR (95% CI) | 1.00 (Ref) | 1.13 (0.99-1.31) | 0.93 (0.69-1.24) | 0.387 | 1.00 (Ref) | 0.97 (0.85-1.10) | 0.99 (0.87-1.13) | 0.971 | 1.00 (Ref) | 1.05 (0.92-1.21) | 1.14 (0.99-1.32) | 0.068 |
|  |  |  |  |  |  |  |  |  |  |  |  |  |
| **Regional/distant metastatic**^¥^ |  |  |  |  |  |  |  |  |  |  |  |  |
| Age-adjusted OR (95% CI) | 1.00 (Ref) | 1.33 (1.14-1.55) | 1.43 (1.06-1.93) | <0.001 | 1.00 (Ref) | 1.02 (0.88-1.17) | 1.06 (0.92-1.22) | 0.433 | 1.00 (Ref) | 0.97 (0.83-1.14) | 1.31 (1.13-1.53) | 0.002 |
| Multivariate adjusted OR (95% CI) | 1.00 (Ref) | 1.22 (1.04-1.42) | 1.10 (0.81-1.49) | 0.043 | 1.00 (Ref) | 0.97 (0.84-1.12) | 0.95 (0.82-1.11) | 0.542 | 1.00 (Ref) | 0.91 (0.78-1.07) | 1.18 (1.00-1.38) | 0.152 |

**Table S2: Odds ratios (OR) and 95% CI for prostate cancer risk categories by baseline levels of serum glucose, total cholesterol and triglycerides for men with measurements more than two years prior to diagnosis.**

- Multivariate models-adjusted for age, educational level, Charlson co-morbidity index, serum glucose, total cholesterol, triglycerides, fasting status, time between measurement and prostate cancer diagnosis.
- *Not adjusted for Charlson co-morbidity index
- Abbreviation: vs= versus, Ref= Reference

**Table S3: Odds ratio and 95% CI for the association between serum lipids (total cholesterol and triglycerides) and glucose levels and PSA levels and Gleason score.**

|  | **Glucose (mmol/l)*** | | |  | **Total cholesterol (mmol/l)** | | |  | **Triglycerides (mmol/l)** | | |  |
| --- | --- | --- | --- | --- | --- | --- | --- | --- | --- | --- | --- | --- |
|  | **<5.60** | **5.60-6.90** | **>6.90** | **P for trend** | **<5.18** | **5.18-6.19** | **>6.19** | **P for trend** | **<1.70** | **1.70-2.24** | **≥2.25** | **P for trend** |
| **PSA < 4 vs 4.0-9.9(µg/l)** | 971 | 152 | 41 |  | 329 | 448 | 387 |  | 816 | 188 | 160 |  |
| Age-adjusted OR (95% CI) | 1.00 (Ref) | 1.06 (0.88-1.28) | 1.41 (1.00-2.01) | 0.081 | 1.00 (Ref) | 0.99 (0.86-1.01) | 0.84 (0.99-1.16) | 0.105 | 1.00 (Ref) | 1.06 (0.89-1.26) | 0.94 (0.78-1.13) | 0.698 |
| Multivariate adjusted OR (95% CI) | 1.00 (Ref) | 1.08 (0.89-1.30) | 1.43 (1.00-2.05) | 0.067 | 1.00 (Ref) | 0.99 (0.85-1.17) | 0.89 (0.75-1.05) | 0.145 | 1.00 (Ref) | 1.09 (0.91-1.30) | 0.98 (0.81-1.19) | 0.914 |
|  |  |  |  |  |  |  |  |  |  |  |  |  |
| **PSA 4.0-9.9 (µg/l)**  (Reference level for outcome studied) |  |  |  |  |  |  |  |  |  |  |  |  |
|  |  |  |  |  |  |  |  |  |  |  |  |  |
| **PSA 10.0-20.0 vs 4.0-9.9(µg/l)** |  |  |  |  |  |  |  |  |  |  |  |  |
| Age-adjusted OR (95% CI) | 1.00 (Ref) | 1.05 (0.92-1.19) | 1.04 (0.80-1.35) | 0.485 | 1.00 (Ref) | 1.09 (0.98-1.22) | 1.03 (0.92-1.16) | 0.754 | 1.00 (Ref) | 1.05 (0.93-1.19) | 1.13 (1.00-1.28) | 0.044 |
| Multivariate-adjusted OR (95% CI) | 1.00 (Ref) | 0.97 (0.85-1.11) | 0.89 (0. 68-1.17) | 0.392 | 1.00 (Ref) | 1.05 (0.94-1.18) | 0.98 (0.87-1.10) | 0.600 | 1.00 (Ref) | 1.03 (0.91-1.17) | 1.11 (0.98-1.26) | 0.118 |
|  |  |  |  |  |  |  |  |  |  |  |  |  |
| **PSA >20.0 vs 4.0-9.9(µg/l)** |  |  |  |  |  |  |  |  |  |  |  |  |
| Age-adjusted OR (95% CI) | 1.00 (Ref) | 1.41 (1.25-1.60) | 1.41 (1.10-1.81) | <0.001 | 1.00 (Ref) | 0.97 (0.86-1.09) | 1.00 (0.89-1.13) | 0.889 | 1.00 (Ref) | 1.01 (0.89-1.14) | 1.22 (1.08-1.38) | 0.004 |
| Multivariate adjusted OR (95% CI) | 1.00 (Ref) | 1.28 (1.13-1.45) | 1.06 (0.82-1.37) | 0.004 | 1.00 (Ref) | 0.92 (0.82-1.04) | 0.91 (0.81-1.03) | 0.173 | 1.00 (Ref) | 0.97 (0.85-1.11) | 1.17 (1.03-1.34) | 0.048 |
| **Gleason ≤6**  (Reference level for outcome studied) |  |  |  |  |  |  |  |  |  |  |  |  |
|  |  |  |  |  |  |  |  |  |  |  |  |  |
| **Gleason 7 vs ≤6** |  |  |  |  |  |  |  |  |  |  |  |  |
| Age-adjusted OR (95% CI) | 1.00 (Ref) | 1.14 (1.02-1.28) | 1.04 (0.81-1.33) | 0.075 | 1.00 (Ref) | 0.95 (0.86-1.06) | 0.97 (0.87-1.07) | 0.581 | 1.00 (Ref) | 1.02 (0.91-1.14) | 1.06 (0.95-1.18) | 0.319 |
| Multivariate adjusted OR (95% CI) | 1.00 (Ref) | 1.18 (1.05-1.33) | 1.09 (0.84-1.40) | 0.017 | 1.00 (Ref) | 0.97 (0.87-1.07) | 0.98 (0.88-1.09) | 0.680 | 1.00 (Ref) | 1.03 (0.92-1.16) | 1.07 (0.95-1.20) | 0.257 |
|  |  |  |  |  |  |  |  |  |  |  |  |  |
| **Gleason ≥8 vs ≤6** |  |  |  |  |  |  |  |  |  |  |  |  |
| Age-adjusted OR (95% CI) | 1.00 (Ref) | 1.27 (1.09-1.46) | 1.39 (1.03-1.87) | <0.001 | 1.00 (Ref) | 1.06 (0.92-1.22) | 1.15 (1.00-1.32) | 0.047 | 1.00 (Ref) | 1.10 (0.95-1.28) | 1.26 (1.10-1.45) | 0.001 |
| Multivariate adjusted OR (95% CI) | 1.00 (Ref) | 1.24 (1.07-1.44) | 1.38 (1.02-0.87) | 0.001 | 1.00 (Ref) | 1.05 (0.92-1.21) | 1.11 (0.96-1.28) | 0.149 | 1.00 (Ref) | 1.08 (0.93-1.25) | 1.21 (1.04-1.41) | 0.012 |
| **T1-T2**  (Reference level for outcome studied) |  |  |  |  |  |  |  |  |  |  |  |  |
|  |  |  |  |  |  |  |  |  |  |  |  |  |
| **T3-T4 vs T1-T2** |  |  |  |  |  |  |  |  |  |  |  |  |
| Age-adjusted OR (95% CI) | 1.00 (Ref) |  |  |  | 1.00 (Ref) |  |  |  | 1.00 (Ref) |  |  |  |
| Multivariate adjusted OR (95% CI) | 1.00 (Ref) | 1.21 (1.08-1.36) | 1.41 (1.13-1.77) | <0.001 | 1.00 (Ref) | 0.98 (0.87-1.09) | 1.04 (0.93-1.16) | 0.380 | 1.00 (Ref) | 1.11 (0.99-1.25) | 1.18 (1.05-1.32) | 0.003 |

- Multivariate models-adjusted for age, educational level, Charlson co-morbidity index, serum glucose, total cholesterol, triglycerides, fasting status, time between measurement and prostate cancer diagnosis.
- *Not adjusted for Charlson co-morbidity index
- Abbreviation: vs= versus, Ref= Reference
